# Sowing, Monitoring, Detecting: A Possible Solution to Improve the Visibility of Cropmarks in Cultivated Fields

**DOI:** 10.3390/jimaging11030071

**Published:** 2025-02-25

**Authors:** Filippo Materazzi

**Affiliations:** Department of Science of Antiquities, Sapienza University of Rome, 00185 Rome, Italy; filippo.materazzi@uniroma1.it

**Keywords:** UAS, UAV, drone, multispectral, remote sensing, cropmarks, vegetation index, archaeology of pre-Roman Italy, ancient urbanism, Falerii

## Abstract

This study explores the integration of UAS-based multispectral remote sensing and targeted agricultural practises to improve cropmark detection in buried archaeological contexts. The research focuses on the Vignale plateau, part of the pre-Roman city of Falerii (Viterbo, Italy), where traditional remote sensing methods face challenges due to complex environmental and archaeological conditions. As part of the Falerii Project at Sapienza Università di Roma, a field was cultivated with barley (*Hordeum vulgare* L.), selected for its characteristics, enabling a controlled experiment to maximise cropmark visibility. The project employed high-density sowing, natural cultivation practises, and monitoring through a weather station and multispectral imaging to observe crop growth and detect anomalies. The results demonstrated enhanced crop uniformity, facilitating the identification and differentiation of cropmarks. Environmental factors, particularly rainfall and temperature, were shown to significantly influence crop development and cropmark formation. This interdisciplinary approach also engaged local stakeholders, including students from the Istituto Agrario Midossi, fostering educational opportunities and community involvement. The study highlights how tailored agricultural strategies, combined with advanced remote sensing technologies, can significantly improve the precision and efficiency of non-invasive archaeological investigations. These findings suggest potential developments for refining the methodology, offering a sustainable and integrative model for future research.

## 1. Introduction

After an initial pioneering phase in the early 2010s [1,2,3,4,5], the number of publications on multispectral and thermal remote sensing using UASs to detect cropmarks and soilmarks related to archaeological buried remains has increased exponentially since 2018 [6,7,8,9,10,11,12], due to the gradual introduction of more affordable and user-friendly UASs, sensors, and software on the market. In recent years, both methodologies have gained widespread adoption globally, becoming integral to non-invasive survey techniques for studying archaeological contexts, particularly large-scale sites. Today, low-altitude remote sensing applications are frequently combined with other methods, especially magnetometry and GPR [13,14,15,16,17], to collect more comprehensive data for cross-referencing and improving interpretation.

UAS-based multispectral remote sensing involves the use of cameras equipped with multiple sensors capable of simultaneously capturing images in different bands of the electromagnetic spectrum, providing diverse types of information about the surveyed area. These sensors typically detect specific wavelength ranges, including at least NIR (750–1400 nm), which is crucial for vegetation analysis together with red and green. Although plant leaves reflect light in the green spectrum, they reflect much more in the NIR range, while absorbing most of the red wavelengths. Thus, a multispectral camera incorporating NIR enables the detection of subtle differences imperceptible to the human eye, which may relate to cellular structure, pigmentation, and, more generally, to the ‘health status’ of plants [18] (pp. 133–137).

These alterations may be caused by buried archaeological remains, leading to the formation of cropmarks. They can be either positive, where more vigorous vegetation develops, or negative, where plants under stress exhibit reduced growth and yellow earlier. In summary, their appearance depends primarily on the plant species present, the associated farming practises, the weather conditions, as well as on the inherent characteristics of the buried materials, their state of preservation, and burial depth. Equally important are the physico-chemical properties of the soil and the various actions by humans or animals that can prevent or complicate their development.

From the earliest experiments, it quickly became evident that the dynamics of cropmark formation required thorough investigation in relation to the numerous parameters involved [1] (pp. 2–3), which has long been known [19] to influence the likelihood of obtaining results. This led to the formulation of an interdisciplinary approach, primarily focused on agronomy, with the aim of improving the success rate of this method by deepening the study of the complex relationship between buried remains and plants [20,21].

Since 2017, studies conducted at the site of Veii (Rome, Italy), within the teachings of Archaeology of Pre-Roman Italy and Etruscology, have enabled the testing of UAS remote sensing through hundreds of repeated flights over an area of approximately 800 hectares. These efforts have provided valuable experience across various environmental contexts, characterised by different crops and spontaneous vegetation [22].

The data obtained over the years confirm the importance of thoroughly understanding and analysing the area under investigation from multiple perspectives. The primary and most critical factors influencing the success of remote sensing are the plant species present and the long-term weather conditions from the time of sowing onwards. Certain crops with homogeneous characteristics, such as wheat (*Triticum* spp.), have proven particularly suitable for observing well-defined cropmarks, while others, such as herbage composed of diverse species, can significantly complicate observations [21].

Agricultural practises also play a significant role in the results. Low-density sowing can increase weeds and reduce crop uniformity; fertilisation and phytosanitary treatments may create heterogeneous areas, while no-till farming often results in highly inconsistent and patchy crops.

As for fields no longer cultivated and left to grow spontaneously, it has been observed that such abandonment, even if temporary, diminishes the chances of detecting cropmarks. In the portion of central Tyrrhenian Italy characterised by the presence of tuff, this phenomenon is partly due to the low productivity of some fields, caused by bedrock surfacing, rising diesel costs, and, above all, the uncontrolled spread of wild boars, which devastate crops and cause significant losses for farmers [23].

Along with these few examples, which reflect a much more complex situation even when soil characteristics are identical, we must also consider the impact of weather. Intense rainfall during critical phenological stages for cropmark formation, or even destructive atmospheric events such as hail and strong winds, can completely ruin ‘promising’ crops [24].

Even from this brief overview, it is evident that the variables involved are numerous, and sometimes one must concede to unfavourable conditions. Unfortunately, when these conditions result from agricultural practises, it is not enough to simply postpone data acquisition to different periods or years in the hope of better seasons. For this reason, a new investigative methodology has been developed. The experimentation is an integral part of the Falerii Project, which began in 2019 under the teaching of Archaeology of pre-Roman Italy at the Sapienza Università di Roma (P.I. Prof. Maria Cristina Biella). The project aims to offer a holistic reconsideration of the pre-Roman city of Falerii (Viterbo, Italy) through an interdisciplinary approach [25].

With the aim of maximising the achievable results through UAS multispectral remote sensing in an area particularly complex for cropmark detection, an innovative project combining archaeology with agriculture was launched in 2023. Due to unsuitable conditions for remote sensing, which nevertheless has proven to be one of the most promising techniques [26], it was decided to intentionally sow a plant species particularly prone to manifesting cropmarks in an area of interest and to monitor plant growth using different techniques, correlating it with meteorological data obtained from a nearby weather station.

The experiments were carried out and will continue in close collaboration with the Soprintendenza Archeologia, Belle Arti e Paesaggio per la provincia di Viterbo e per l’Etruria Meridionale, as well as with the Municipality of Civita Castellana (Viterbo).

Through this non-invasive, multi-temporal, and interdisciplinary experimentation, new data were obtained for the understanding of a vast area that is impossible to fully investigate through excavation, at least in the near future. It also allows for a better understanding of the manifestation and development patterns of cropmarks based on environmental conditions and buried remains. Consequently, we can enhance our ability to interpret the data, providing important clues for expanding the investigation through stratigraphic excavation.

The project also involved the participation of the local upper secondary school Istituto Agrario Midossi, which facilitated students’ engagement with archaeological research on one hand and UAV-based multispectral remote sensing on the other, the learning of which is also beneficial for their professional curriculum.

## 2. Materials and Methods

### 2.1. Case Study

The field chosen for the experiment is located in Civita Castellana (Viterbo, Italy), more specifically on the smaller plateau of the pre-Roman city of Falerii.

**Figure 1:**
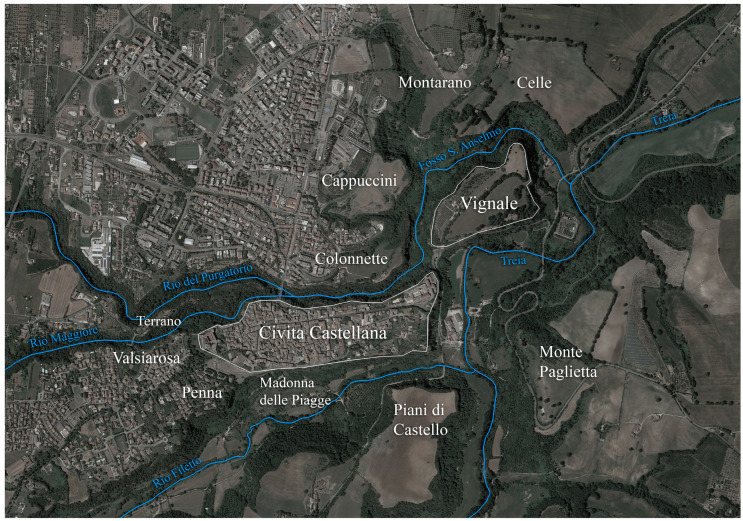
The two plateaux of Falerii (Civita Castellana and Vignale) and the surrounding hills.

Falerii is approximately 50 km north of Rome, in the middle Tiber Valley. The site is situated in a landscape of volcanic origin, characterised by tuff plateaux and deep ravines (Figure 1). The pre-Roman settlement was located on two hills: the so-called Vignale and the Civita Castellana hill, which together cover an area of about 44 ha. According to the ancient written sources, Falerii was abandoned in 241 BC, following the Roman military conquest, that led also to the foundation of a new city: Falerii Novi (today Fabrica di Roma). After its fall, the former remained uninhabited and was used primarily for pastoral purposes and, to a lesser extent, agriculture, while the larger hill is now occupied by the Medieval and modern town of Civita Castellana [27].

For this reason, Vignale seemed an exceptional case study and has therefore been systematically and repeatedly surveyed both on the ground and from the air. Since 2022, a large area has been under excavation, using the results of the UAS remote sensing as a starting point [26] (pp. 4–6), integrated with historical aerial photographs, a new GPR campaign [25] (pp. 93–96), and the outcomes of the magnetometry and LiDAR surveys conducted by the British School at Rome at the end of the last century as part of the Roman Towns in the Middle and Lower Tiber Valley Project [28].

**Figure 2:**
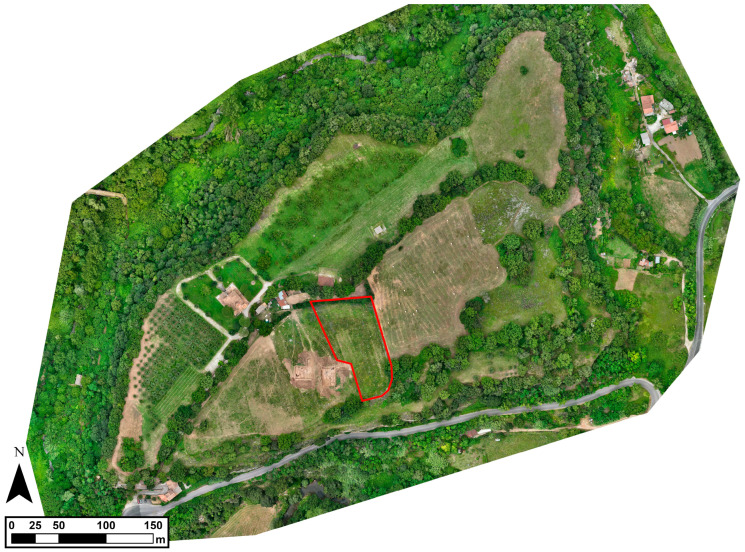
Ortophoto of the Vignale hill (29 June 2023). The red outline indicates the perimeter of the test field (42.293786, 12.424435).

The Vignale plateau covers an area of about 14 ha and has an elongated shape sloping in a northeasterly direction (Figure 2). It includes hazelnut and olive trees, meadows, and fields cultivated as herbage, one of which has been the focus of the research and excavation campaigns.

Thus, the situation at Vignale is notably complex from both environmental and archaeological perspectives. The field under study was typically cultivated with low-density herbage for hay production, comprising a mixture of herbaceous species with different requirements and varying stages of development. This diversity, in which underdeveloped and end-of-life individuals grow in close proximity to one another, prevents the formation of well-defined cropmarks, particularly those caused by buried remains of small dimensions. Moreover, the depth at which these remains are buried may vary across different areas of the hill, depending on the thickness of the soil layer overlying the bedrock. To overcome this critical factor in cropmark formation, the plants must have deep-rooting systems capable of reaching significant depths [26] (pp. 4–7). For these reasons, both satellite imagery and UAS-based remote sensing have identified only a few anomalies, most of which are related to post-ancient activities [25] (pp. 88–93).

### 2.2. Farming Practises

The field chosen for the experimental cultivation is located immediately east of the two excavation trenches, in the southern part of the Vignale plateau, covering an area of approximately half a hectare (Figure 2). The field has an elongated shape, is oriented north–south, and gently slopes southward toward the edge of the plateau. From an archaeological perspective, the area is almost entirely unexplored, as previous investigations have not yielded significant results. However, its proximity to the excavation area makes obtaining information about it particularly valuable.

To recreate the ideal conditions for cropmark formation, specific measures were taken, starting with the choice of crop. In our case, barley (*Hordeum vulgare* L. *var. Gemini*) was selected. Like wheat (*Triticum* spp.), it is predominantly a self-pollinating species, which ensures that the crop consists of genetically similar individuals that develop as uniformly as possible, guaranteeing a homogeneous crop. Therefore, any differences in plant growth are theoretically attributable to soil characteristics and variations in weather conditions [21] (pp. 14–18).

Furthermore, this species, like wheat, has a fasciculated root system, which can extend vertically up to 1.3 m, potentially allowing it to reach deeper remains and thus, improve the investigation of the subsoil [29] (pp. 351–352). The cultivation is managed to avoid artificially altering plant growth, aiming to maximise the possibility of identifying cropmarks.

**Figure 3:**
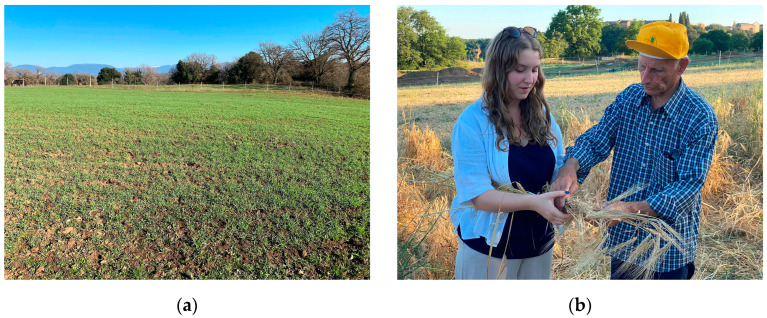
(**a**) Barley growing in the test field (5 January 2024). (**b**) Sapienza student learning traditional barley harvesting techniques.

The field was not ploughed to avoid damaging the archaeological deposit; instead, it was mulched and worked to a depth of 15–20 cm, within the limits imposed by law, using a disc harrow to prepare it for sowing. This took place on 25 November 2023, with a sowing density of 220 kg/ha, aimed at reducing weeds and improving crop uniformity, with the goal of identifying even the smallest buried remains (Figure 3a). To prevent altering vegetative development, no products of any kind were used, adhering to a completely natural cultivation approach.

The barley reached full maturity in May and was harvested in June. During this initial year of research, the majority of the yield was utilised as animal fodder, primarily due to the logistical challenges of navigating a combine harvester through the narrow access paths to the Vignale hill. A smaller portion of the crop was harvested manually using traditional techniques as part of an experimental archaeology approach (Figure 3b).

### 2.3. Monitoring

**Figure 4:**
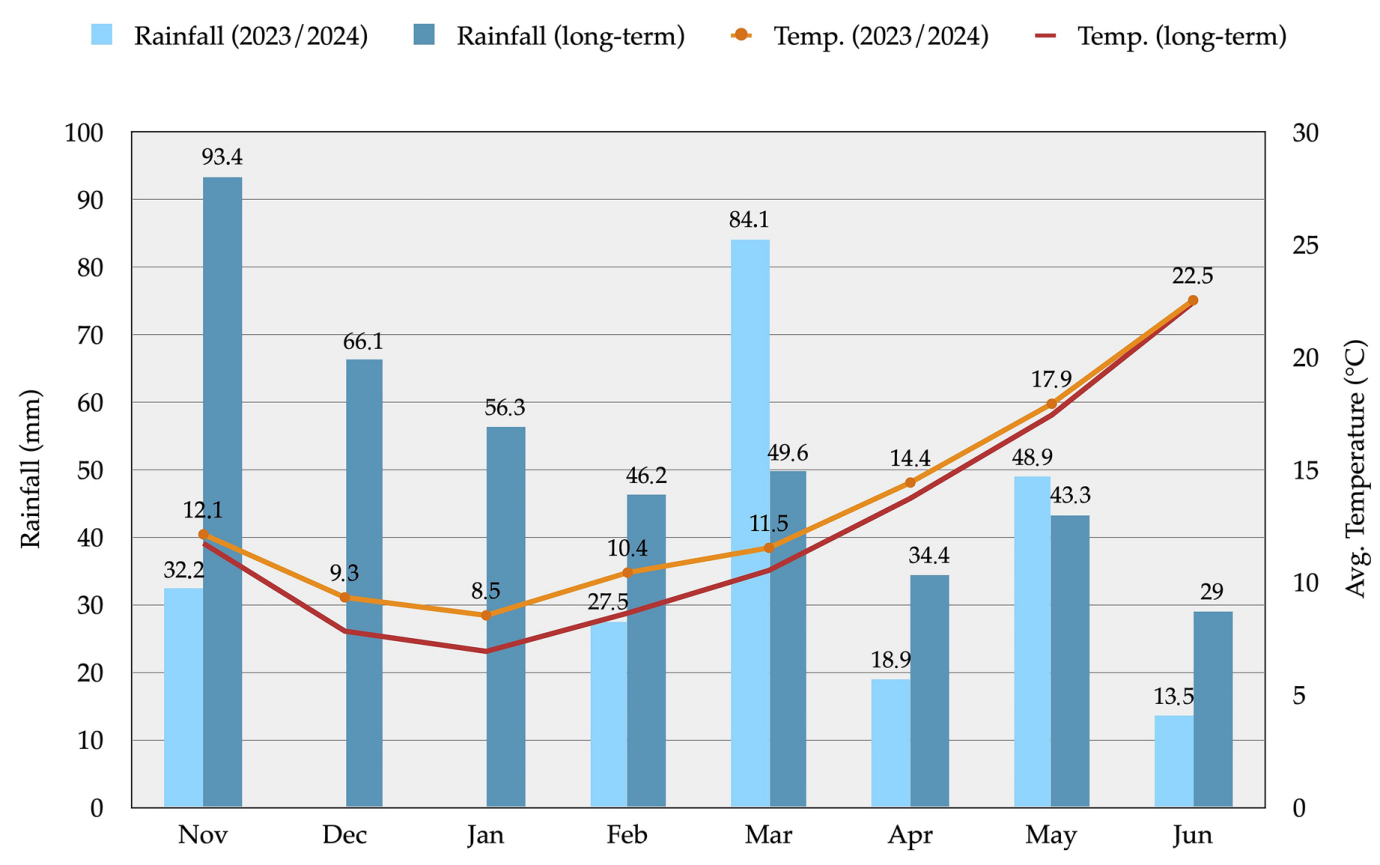
Histogram of rainfall and air temperature values recorded by the weather station.

Between November and May, the cultivation was monitored through direct observation to assess the phenology of the plants, growth uniformity, and the extent of weed infestation in relation to seasonal weather conditions. Meteorological data were analysed from the nearest available weather station, located 140 m above sea level near the modern town of Civita Castellana, approximately 1.5 km from the study area. Particular attention was paid to rainfall and temperature starting from the time of sowing, comparing them to the monthly averages of previous years, as well as to intense events such as thunderstorms and hail that could potentially damage the crop (Figure 4).

For UAS monitoring, the DJI Mavic 3M was used; a compact, foldable, and easily transportable quadcopter equipped with a multispectral camera featuring a radiometric calibration sensor and GNSS RTK. The imaging system includes a 20 MP RGB camera with a 4/3" sensor and four single-band 5 MP sensors: green (560 ± 16 nm), red (650 ± 16 nm), red-edge (730 ± 16 nm), and NIR (860 ± 26 nm). The effective flight time was 30 min, which allowed coverage of up to 50 hectares without overstraining the batteries.

The flight mission was planned using the DJI Fly app installed on the UAV controller, ensuring the same characteristics for all acquisitions and making the images perfectly comparable. Flights were conducted under consistent lighting conditions and calm winds during midday hours to reduce shadows and improve the reliability of reflectance data. Automated flights were planned at a constant altitude of 50 m above ground level, ensured by the UAV’s real-time terrain awareness feature, at a speed of 2.7 m/s, with 80% front overlap, and 75% side overlap. This configuration yielded single-band images with a GSD of 2.3 cm/pixel and RGB images with a GSD of 1.3 cm/pixel. Radiometric calibration relied on the onboard light sensor without using a ground calibration panel, while georeferencing was achieved solely with GNSS RTK, providing sufficient accuracy for orthoimage georeferencing to meet the project’s objectives.

Flights were conducted according to environmental conditions, as previously described, on 12 January, 15 March, 5 April, 11 May, and 17 May 2024.

### 2.4. Data Processing

**Scheme 1:**
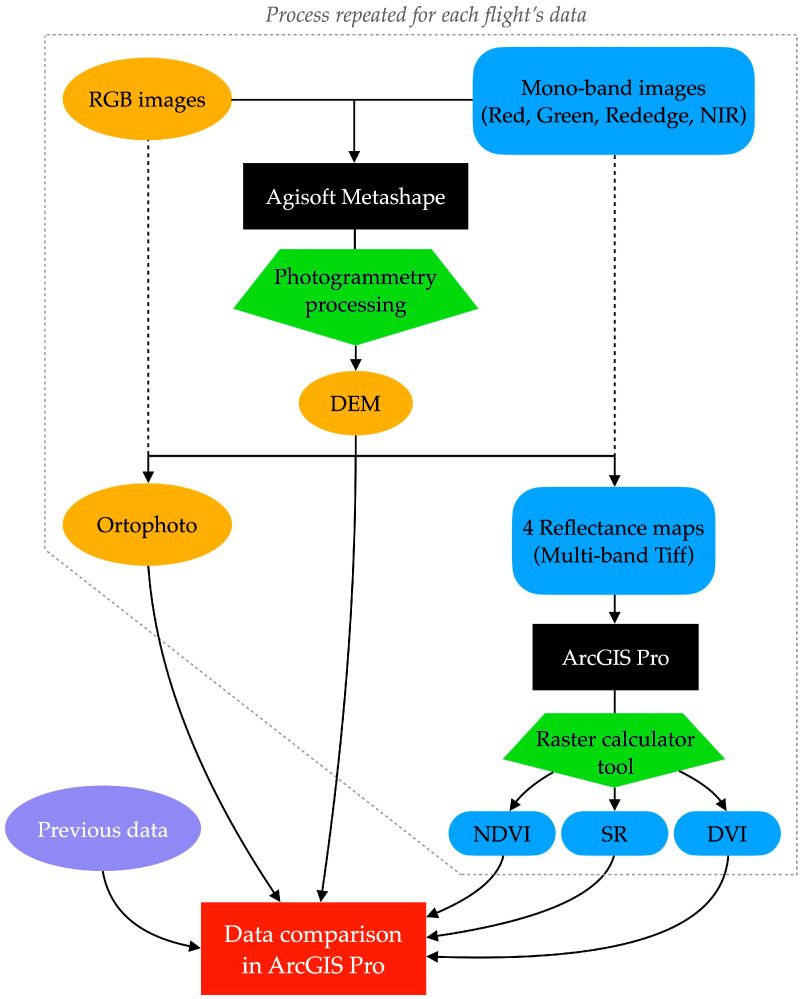
Diagram of the image processing.

For data processing, the photogrammetry software Agisoft Metashape Professional 2.1.1 was used (Scheme 1). All images from each individual flight were processed simultaneously to generate aligned maps with perfectly overlapping pixels. To obtain absolute reflectance values, radiometric calibration was performed before starting the process, using the light sensor values associated with each image. In addition to the DEM and RGB orthophoto, a multi-band TIFF file was exported containing the reflectance maps derived from the single-band images [21] (p. 10).

The maps were then imported into ArcGIS Pro for the second phase of processing (Scheme 1). Reflectance maps were analysed using mathematical formulas known as spectral indices. In agronomy, these formulas are referred to as vegetation indices and are used to indirectly monitor various plant characteristics, such as vigour, biomass, leaf pigments, and more [30]. In this study, the primary index employed was the NDVI (Equation (1)) [31], a widely used index in the literature [32].

(1) $$NDVI=\frac{NIR-RED}{NIR+RED}$$

NDVI relates spectral absorption in the red band to reflection in the NIR band to estimate plant vigour. Being normalised, it allows for an immediate comparison of vegetation on a value scale ranging from −1 to 1. For vegetation, NDVI values are always positive, typically ranging between 0.3 and 1, while values near 0 correspond to bare soil.

For the 11 May image set, acquired during the period of maximum intra-field differences, which will be shown later, two additional vegetation indices were applied in an attempt to improve cropmark reading: SR (Equation (2)) and DVI (Equation (3)).

(2) $$SR=\frac{NIR}{RED}$$

(3) DVI=NIR−RED

These indices, while relatively simple, have proven effective in identifying cropmarks under similar environmental conditions [22].

**Figure 5:**
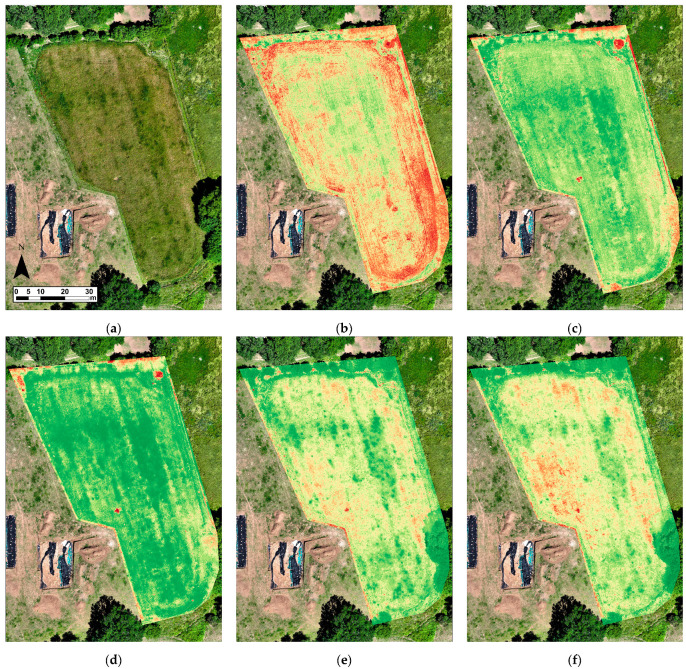
RGB and NDVI maps with red-yellow-green colour scale associated with a linear value scale ranging from 0.3 (red) to 1 (green): (**a**) RGB orthomap 11 May 2024, (**b**) NDVI 12 January 2024, (**c**) NDVI 15 March 2024, (**d**) NDVI 5 April 2024, (**e**) NDVI 11 May 2024, and (**f**) NDVI 17 May 2024.

**Figure 6:**
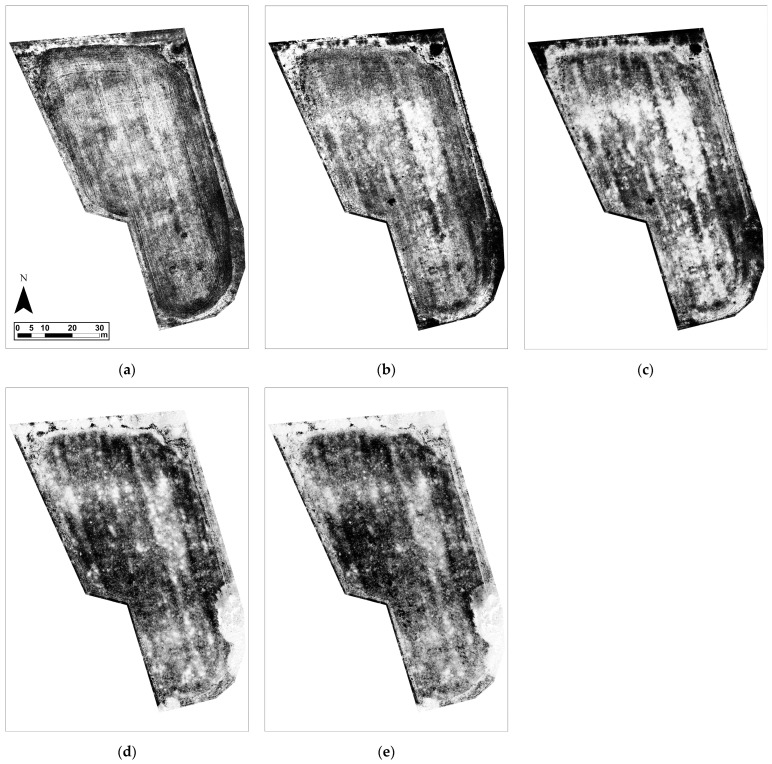
NDVI maps with enhanced contrast: (**a**) NDVI 12 January 2024, (**b**) NDVI 15 March 2024, (**c**) NDVI 5 April 2024, (**d**) NDVI 11 May 2024, and (**e**) NDVI 17 May 2024.

**Figure 7:**
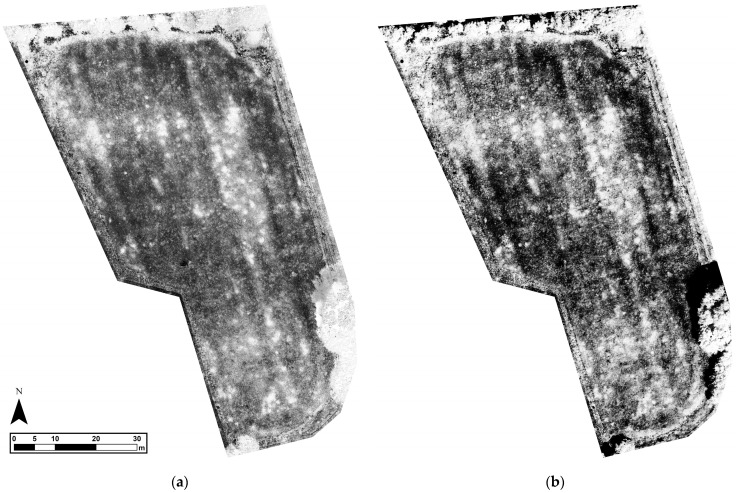
Index maps of 11 May 2024 with enhanced contrast: (**a**) SR and (**b**) DVI.

The index maps were compared within the GIS to analyse vegetative development and cross-referenced with other available data, including the RGB orthophoto, to expand knowledge of the studied area. To compare vegetative vigour, NDVI maps were exported using a red-yellow-green colour scale associated with a linear value scale ranging from 0.3 (red) to 1 (green) (Figure 5). To facilitate the identification of anomalies, the maps were enhanced using the “Histogram Equalize” stretch type in ArcGIS to increase contrast and applying a black–white scale (Figure 6 and Figure 7). Finally, the identified cropmarks were digitised using vector polygons to create a comprehensive map of features. The reading of cropmarks, their interpretation, and ultimately the digitisation was carried out manually, relying on the experience accumulated over the years. It was deemed impossible to approach the task automatically in such a complex context, where the correlation between different data and local knowledge proved essential for identifying the cropmarks.

Therefore, cropmarks were identified by first observing anomalies with higher index values, due to plants with greater chlorophyll content and biomass produced by ditches, pits, and depressions in the bedrock or by tuff structures, a porous stone that retains moisture. Secondly, the shape and dimensional characteristics of the anomalies were taken into account, distinguishing them within the context in which they are located.

### 2.5. Previous Data

**Figure 8:**
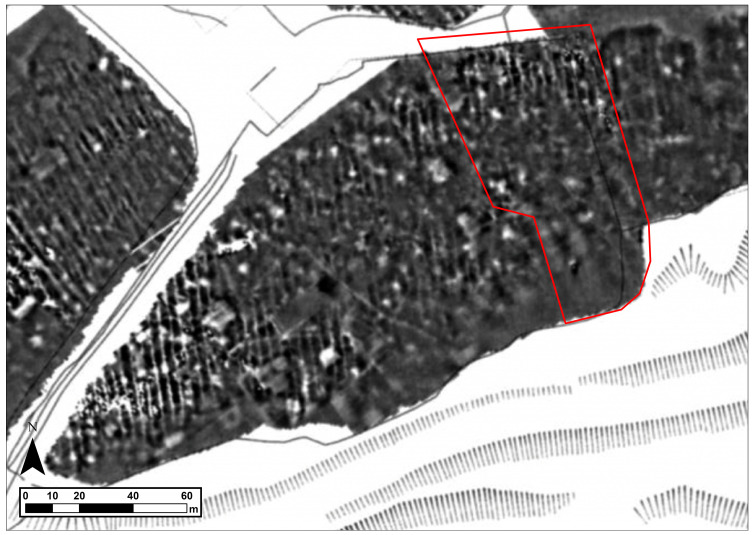
Magnetometry results from the southern section of the Vignale plateau (2001–2002) [28]. The red outline indicates the perimeter of the test field.

Additionally, other data were available for comparison with the new acquisitions (Scheme 1). Among these were the results of an acquisition using a fluxgate magnetometer by the British School at Rome, conducted across the Vignale plateau (Figure 8) [28]. This survey revealed that the entire area had been cultivated with vineyards, as suggested by the name ‘Vignale’, prior to the 20th century, as evidenced by archival research [25] (pp. 90–92). To facilitate the planting of the vines, the tuff bedrock was excavated in some areas due to its shallow burial, which severely damaged the stratigraphic deposit at regular intervals, creating trenches approximately 80 cm wide and spaced about 3 m apart, as confirmed by archaeological excavation. Despite this, the magnetometry identified several anomalies corresponding to structures with varying characteristics, especially in the southern half of the plateau.

The test field was also only marginally included in a GPR campaign conducted in July 2021 over 6800 m^2^, which did not yield particularly clear results due to the low contrast caused by the exclusive presence of tuff structures resting on the bedrock of the same material [25] (pp. 93–96).

**Figure 9:**
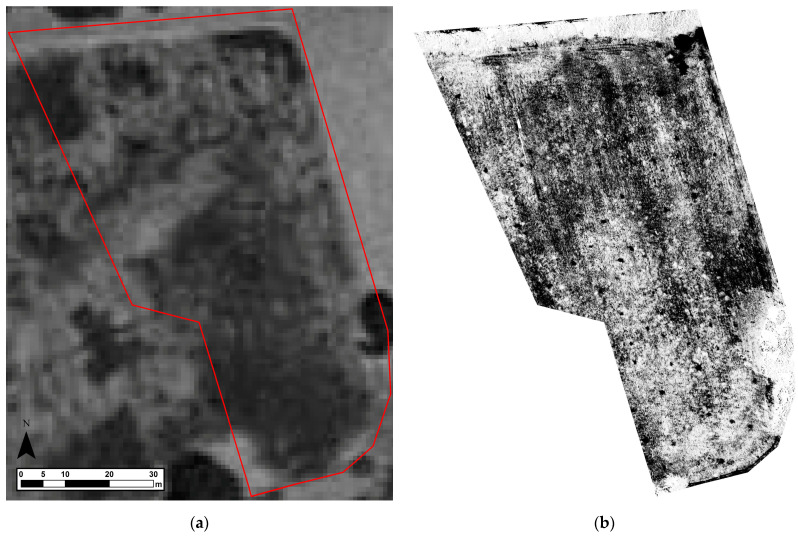
(**a**) Aerial photo (August 1996) [33]. The red outline indicates the perimeter of the test field. (**b**) NDVI (15 May 2021).

A particularly useful aerial image from August 1996, available on the Italian National Geoportal [33], shows that the south-western field was likely cultivated with alfalfa (*Medicago sativa* L.) (Figure 9a). The vegetation that grew during the summer season formed a series of fairly defined cropmarks, which largely coincide with the anomalies detected by magnetometry and, in some cases, help clarify them. Unfortunately, the low resolution of the image limits the detection to only the larger features. Despite these findings, neither method provides clearly interpretable evidence in the test field. Magnetometry, except for a small portion in the northern part of the field, indicates the absence of vine trenches, likely because the bedrock was sufficiently deep. The lush vegetation growth, particularly in the central-eastern and southern parts of the field, visible in the 1996 image, seems to support this interpretation.

Within the Falerii Project, some UAS flights using multispectral and thermal cameras were previously conducted to investigate the Vignale plateau and the surrounding hills [25] (pp. 88–93). The most significant results for the test field area were obtained from the multispectral acquisitions of 15 May 2021. Despite the herbage cultivation, the vegetation indices provided additional information, which helped guide the positioning of the excavation trenches. In this context, the NDVI map with the ‘Histogram Equalize’ stretch type from ArcGIS Pro, using a black–white scale, is presented (Figure 9b).

**Figure 10:**
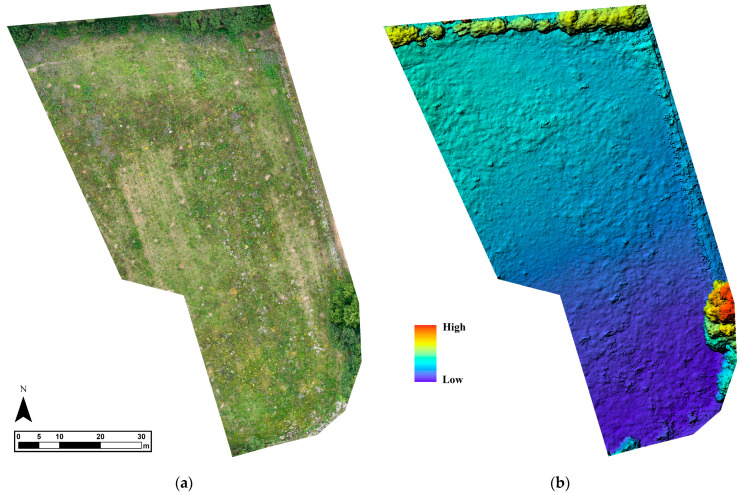
Flights of 29 June 2023. (**a**) Ortophoto and (**b**) DEM.

Additionally, there are UAS data from 29 June 2023, where nadiral images were supplemented with oblique ones to capture the entire Vignale plateau with greater geometric accuracy, producing an orthophoto (Figure 10a), a DEM (Figure 10b), and a high-resolution 3D model.

## 3. Results

### 3.1. Cultivating Barley

**Figure 11:**
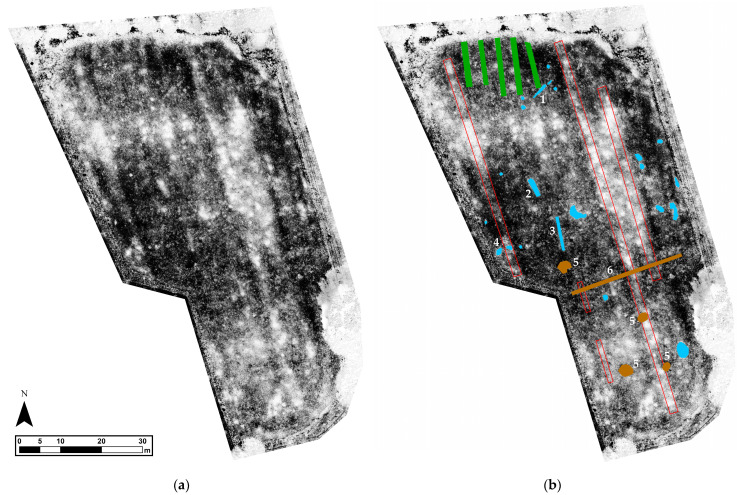
(**a**) NDVI of 11 May 2024 with enhanced contrast. (**b**) Digitised cropmarks. Green: post-antique vineyard trenches; red: post-antique shallow ditches; brown: false marks made by animals; blue: potential archaeological marks.

Despite the superficial cultivation of the soil, a relatively homogeneous crop was achieved, which proved to be much more suitable for cropmark detection compared to the previous herbage. As expected, weeds were present despite the high-density sowing, but they appeared in limited quantities and were evenly distributed, meaning they did not pose a significant issue. The exception was at the margins of the test field, where spontaneous herbs outcompeted the barley, thereby reducing the area effectively available for identifying features. This was further exacerbated by less dense sowing in these areas. Additionally, damage caused by wild boar, which dug in search of food at four spots across the field, hindered crop growth. These disturbances occurred three times prior to the flight on 12 January and once before the flight on 15 March (Figure 5 and Figure 11: 5).

These events were easily identified both from the ground and aerial observations, particularly using RGB photography, but only because the field was monitored shortly after the disturbances. Later, these gaps were partially filled by spontaneous herbs, which could have been misinterpreted as cropmarks. Consequently, the monitoring proved crucial for distinguishing false marks.

Upon closer examination of the various stages of growth, it was significantly influenced by the prevailing weather conditions. Fortunately, no extreme weather events occurred that could have damaged the crop or caused lodging. However, there were considerable variations in rainfall. After sowing, an anomalous drought persisted until 23 February, which, as shown in the map from 12 January (Figure 5), slowed the growth of the barley. This situation, characterised by only 27.5 mm of rainfall in February and temperatures averaging almost two degrees higher than the monthly norm, contrasted sharply with March, when rainfall (84.1 mm) was nearly twice the monthly average, leading to a surge in plant growth. This is clearly evident in the map from 15 March (Figure 5), where only the south-eastern portion of the field exhibited slower growth due to shading from trees located just beyond the field. By 5 April, the barley had reached approximately its peak vigour (Figure 5). Rainfall continued throughout the second half and end of April (total: 18.9 mm), and especially at the beginning of May (total: 48.9 mm), which contributed to the spread of weeds, partly due to rising temperatures consistent with the season and the delayed maturation of the barley. The maps from 11 and 15 May reveal the progressive yellowing of the crop, following a trend that was partially evident during the growth phase, where the central-eastern portion of the test field exhibited early growth followed by delayed maturation (Figure 5).

### 3.2. Identified Cropmarks

The first result obtained from the analysis of crop vigour is a better understanding of the morphology of the bedrock, thanks to the observation of barley growth. Already in the January flight, and even more so in the March flight, it is evident that there is a fairly clear boundary within the field, between the first 18 m to the north, which exhibits shallow burial, and the southern part where the bedrock deepens (Figure 6). This distinction can also be partially observed in the orthophoto from 29 June 2023 (Figure 10a), when spontaneous grassland was present, and even more clearly in the magnetometry (Figure 8). In the latter, vineyard trenches are visible only at the northern edge of the field, indicating that the bedrock had to be broken due to the shallow soil depth.

Further south, the bedrock appears to maintain its depth, but in a large section to the west, there may be another area with shallow burial. This area is near the eastern trench of the Sapienza excavations, where an underground cuniculus was found, partially uncovered due to the removal of the top layer of the bedrock. This could potentially be the result of a modern excavation carried out using mechanical means during the 1950s–1960s, which would have removed the older trenches associated with vineyard planting. This would explain why these trenches are not visible in the analyses. Alternatively, though less likely, it might reflect a deepening of the bedrock, possibly linked to an ancient, terraced configuration of the landscape. A much larger terracing system, this time oriented to the east, has previously been proposed for the eastern part of the plateau [28] (p. 98).

With the exception of the four false negative cropmarks caused by wild boar holes (Figure 11: 5) and a line likely due to a path created by wild animals (Figure 11: 6), the identified cropmarks are exclusively positive type, i.e., formed by vegetation with high vigour. These are characteristic of the pre-Roman period in areas dominated by tuff. They can form in three ways: due to the presence of ditches and pits, some even dug into the bedrock; due to an accumulation of nutrients potentially linked to human activity; and due to the presence of tuff block walls, a material that retains moisture and delays the senescence process [21] (pp. 22–24). These stone walls began to be built, according to scholarship, no earlier than the second half of the 7th century BCE, and more commonly during the 6th–5th centuries BCE [27].

Positive cropmarks are mainly identified during the later phenological stages, especially during the milk, dough, and ripening phases, when barley, after flowering, completes its development at different rates [21] (pp. 14–18). For this reason, the maps where the cropmarks are most evident are those from April and May, particularly from 11 May, as by 17 May the more advanced yellowing began to equalise the intra-field variations (Figure 6).

The first cropmarks that stand out, in addition to those already presented, are three parallel strips nearly as long as the entire field and a fourth, identifiable only partially, oriented north-northwest/south-southeast (Figure 11, red). These have already been partly identified in the past with previous UAV flights and interpreted as old shallow ditches used to delimit the crops, which do not affect the bedrock and therefore are not detected by magnetometry [25] (pp. 90–93). Less defined, but still recognisable, especially through comparison with the magnetometer data, are five parallel lines approximately 1 m wide and spaced 2.8–3 m apart, produced by the excavations for the vineyard planting at the northern edge of the field (Figure 11: green).

The potential archaeological marks are few and characterised by indistinct shapes, except for a few exceptions. Only the clearest cropmarks present in areas with shallow burial and those that appear in more than one acquisition made in different months were chosen to minimise interpretation errors.

Small, roundish pits of about 70 cm appear scattered across the field, standing out in areas with reduced vigour, and do not seem to follow any particular order (Figure 11). Only in three cases are elongated cropmarks found that differ from the others due to their more likely anthropogenic origin. These are disconnected from one another and exhibit different orientations. Cropmark 1 is located to the north of the field, oriented northeast/southwest, and measures 4.4 m in length, 6 m if a second, slightly offset segment is included, and is 40–70 cm wide. Further south, isolated cropmark 2 measures approximately 4.7 × 1 m, with blurred edges. About 6 m south, cropmark 3 has a similar orientation, is about 70 cm wide, and measures 5.5 m in length when considering the June flights. In the previous month, it appeared over 9 m long, but this seems to be caused by its connection to a mark produced by one of the ditches described earlier, which runs nearly in line with it and distorts its geometry. As the barley ripens, the cropmarks of the ditches, caused by a modest accumulation of moisture, tend to disappear, leaving only the more intense cropmarks, such as in the case of cropmark 3.

These three cropmarks, due to their characteristics, are the only ones that could be attributed to tuff block walls. However, their isolation and the poor definition of their edges make this assumption particularly problematic.

Other cropmarks are also recorded with very blurred edges and more complex, rounded geometries that are not easily interpreted but do not appear to be associated with structures (Figure 11). A number of these are found at the southern edge of the field, but the likely depth of burial in this area raises doubt on their association with buried features, and therefore, they have been partially digitised. Finally, cropmark 4, formed by a blurred patch with two more intense points, seems to correspond to an anomaly identified through magnetometry, which confirms its highly irregular shape.

### 3.3. Closing the Cycle

The cultivation of barley was not solely for archaeological purposes. From our perspective, it is important that this activity also yields results from an educational standpoint, while integrating with traditional agricultural practises and the local production system without altering it but supporting it.

The harvest resulting from this cultivation necessitates careful consideration of its use, focusing on process optimisation and waste elimination. A small portion of the harvest was delivered to the local upper secondary school “Istituto Agrario Midossi”, which specialises in agricultural studies, to experimentally produce a limited-edition beer. This served not only to promote the ongoing archaeological activities but also, and more importantly, to reconnect the local population with its past.

Additionally, the students monitored the crop’s growth as a field-based educational activity, while in the classroom, they attended lessons on the pre-Roman history of the area and the use of UAS remote sensing in agriculture and archaeology. Finally, another class from the same Institute, specialising in audiovisual and multimedia studies, contributed as an integral part of their learning plan by producing a documentary designed to highlight the key themes of the project.

## 4. Discussion

The case study represents a successful, albeit preliminary, experiment demonstrating that the targeted cultivation of a plant species with homogeneous characteristics and a propensity for cropmark manifestation, within a controlled context, can enhance our understanding without disrupting land-use dynamics. Instead, it fosters engagement with the local population, particularly younger generations, by addressing the interrelationship between environment, archaeology, and agricultural production in a region intrinsically connected to these themes. This approach underscores the potential for integrating activities with differing purposes, achieving diverse objectives through a project of optimal efficiency, where the demands of non-invasive archaeological surveys are seamlessly incorporated into the agricultural cycle and further ‘amplified’ by meticulous farming practises.

Although the variables influencing the formation of cropmarks are numerous and often unpredictable—such as those related to the characteristics of buried remains—this study provides valuable insight into the key role of certain factors. Without considering soil characteristics, which remained constant in the case under consideration due to the presence of only a test field, one of the key factors for the formation of well-defined cropmarks is the crop’s properties, particularly its uniformity. Barley was chosen not only for its ‘remote sensing compatibility’, similar to that of wheat [21], but also because, in our specific case, its harvest could be more effectively utilised as fodder and for beer production, avoiding waste. Nevertheless, other crops could also yield excellent results. Specifically, alfalfa (*Medicago sativa* L.) has demonstrated significant efficacy in similar contexts [29] (p. 351), but it would involve higher costs and a longer project timeframe, as it is typically cultivated for at least three years.

Another determining factor for the formation of cropmarks is the weather conditions, as they have a profound impact on crop growth and their competition with invasive species. In this study, it has been observed that the absence of rainfall during the initial months delayed growth and, consequently, the development of the root system, which may not have reached the optimal depth necessary to detect deeper archaeological features, as previously assessed [26] (pp. 6–7). On the other hand, the substantial rainfall in March likely promoted uniform ripening by providing widespread areas of the field with adequate moisture to endure the rising temperatures of May. This condition may have led to the fading of some cropmarks, resulting in poorly defined outlines (Figure 6). In retrospect, a drier May would have been more conducive to the formation of distinct, positive cropmarks, thereby facilitating their interpretation, as the lack of water limits plant growth and accelerates senescence and yellowing, as highlighted in the past in a similar context [21] (pp. 17–18).

To address climate-related challenges, experimenting with the cultivation of other plant species or different varieties within the same species could provide valuable insights, as these may respond differently depending on buried features and climatic conditions. However, a potentially more effective approach to mitigating these issues could involve sowing and analysing perennial crops with different growth cycles that extend into the summer months. In Mediterranean climates, where summer rainfall is minimal or almost entirely absent, such crops are less likely to be affected by precipitation, which could otherwise alter results. Among these, alfalfa holds a particular promise due to its ability to regenerate during July and August, concentrating growth in areas of higher moisture even after minimal rainfall and despite temperatures exceeding 30 °C. This is attributable to its deep taproot system, which can extend beyond 2 m [34], usually delineating buried features with remarkable precision [35] (p. 173). These considerations are especially relevant for positive cropmarks, highlighting the importance of selecting crops and varieties based on the type of features that are expected for observation.

Another strategy to enhance the likelihood of success and simplify the identification and interpretation of data involves repeating aerial surveys over multiple years to mitigate seasonal variability. This approach has proven effective in other contexts [21] (pp. 18–20), and it is evident that continued efforts in this direction should be pursued when possible.

The application of multiple vegetation indices may also aid in clarifying certain cropmarks and, occasionally, in identifying new ones. However, this appears to be particularly beneficial in more complex and less homogeneous vegetative scenarios, such as those involving mixed species [20] (pp. 7–8).

The methodology tested thus far will continue in the 2025 season with a new barley sowing conducted on 30 December 2024 in a western area of the plateau, where magnetometry has identified potential masonry structures. Additionally, UAS-based thermographic monitoring is planned for 2025 to evaluate the crop’s water status and the cropmarks associated with it, as well as to explore the method’s application on bare soil in the search for soilmarks arising from temperature and moisture differences [36].

In the future, these activities could lead to stratigraphic investigations of particularly promising contexts to expand excavations in alignment with the scientific objectives of the project, which primarily focus on the study of settlement dynamics within the pre-Roman habitation. Plans are also in place to extend this experimental approach to other sites, including the Etruscan and Roman city of Veii, which has been the subject of intensive UAS-based remote sensing campaigns since 2017 [22].

## 5. Conclusions

The research presented here marks only the beginning of a new avenue of study in the development of multispectral UAS-based methodologies for investigating buried archaeological contexts. This approach could have the potential to usher in a new phase of research characterised by pronounced interdisciplinarity, requiring collaboration with scholars from diverse fields. Advancing this research entails deepening our understanding of the characteristics of plant species and their varieties in relation to weather and climatic conditions, as well as their sensitivity to stressors caused by buried archaeological features, while also considering other factors influencing the formation of cropmarks [24] (pp. 191–192). Hypotheses must also be rigorously tested through field experiments conducted in controlled conditions wherever possible, as exemplified in the present study. Such efforts could lead to a radical evolution in the potential applications of multispectral UAS remote sensing and enhance our ability to apply this technique with increasing precision, ultimately yielding better and more consistent results.

Certainly, developing the methodology in this direction necessitates extended investigation times and a reduction in the area that can be surveyed. The associated agricultural and monitoring activities, which are both time-consuming and costly, further complicate its application and require full control over the study area, including active collaboration with landowners and any tenants. However, these stakeholders could benefit from the harvest, offering a potential incentive for their cooperation.

This line of research is expected to have a dual impact on multispectral methodology: on the one hand, it will foster greater awareness in the application of the method and the interpretation of results under varying conditions; on the other hand, it will aid in developing an approach that leverages the creation of an ideal agricultural context to tackle complex scenarios, both archaeologically and vegetationally.

## Data Availability

No original images or raw data will be made available on the locations, as they concern archaeological sites.

## Abbreviations

The following abbreviations are used in this manuscript:

UAS	Unmanned Serial System
GPR	Ground Penetrating Radar
NIR	Near Infra-Red
LiDAR	Light Detection And Ranging
RTK	Real Time Kinematic
UAV	Unmanned Aerial Vehicle
GSD	Ground Sampling Distance
DEM	Digital Elevation Model
NDVI	Normalised Difference Vegetation Index
SR	Simple Ratio
DVI	Difference Vegetation Index
GIS	Geographic Information System

## References

[1] Fiorini L., Materazzi F. (2017). Un Iseion a Gravisca? Fotogrammetria, telerilevamento multispettrale da APR e dati archeologici per una possibile identificazione. FOLD&R FastiOnLine Doc. Res..

[2] Casana J., Kntner J., Wiewel A., Cothren J. (2014). Archaeological aerial thermography: A case study at the Chaco-era Blue J community, New Mexico. J. Archaeol. Sci..

[3] Uribe Agudo P., Angás J., Pérez-Cabello F., De La Riva J., Bea M., Serreta A., Magallón M.A., Sáenz C., Martín-Bueno M. Aerial Mapping and Multi-sensors Approaches from Remote Sensing Applied to the Roman Archaeological Heritage. Proceedings of the International Archives of the Photogrammetry, Remote Sensing and Spatial Information Sciences.

[4] Lehmann J.R.K., Smithson K.Z., Prinz T. (2015). Making the Invisible Visible: Using UAS-based High-resolution Color-infrared Imagery to Identify Buried Medieval Monastery Walls. J. Unmanned Veh. Syst..

[5] Šedina J., Housarová E., Matoušková E. (2016). Documentation of Urn Graves of Knovíz Culture by RPAS. Geoinformatics FCE CTU.

[6] Cowley D.C., Cowley D.C., Moriarty C., Geddes G., Brown G.L., Wade T., Nichol C.J. (2018). UAVs in Context: Archaeological Airborne Recording in a National Body of Survey and Record. Drones.

[7] McLeester M., Casana J., Schurr M.R., Hill A.C., Wheeler J.H. (2018). Detecting Prehistoric Landscape Features Using Thermal, Multispectral, and Historical Imagery Analysis at Midewin National Tallgrass Prairie, Illinois. J. Archaeol. Sci. Rep..

[8] Koucká L., Kopačková V., Fárová K., Gojda M. UAV Mapping of an Archaeological Site Using RGB and NIR High-Resolution Data. Proceedings of the 2nd International Electronic Conference on Remote Sensing.

[9] Uribe Agudo P., Angás Pajas J., Pérez-Cabello F., Vicente Redón J., Ezquerra Lebrón B. (2018). The Potential of Drones and Sensors to Enhance Detection of Archaeological Cropmarks: A Comparative Study Between Multi-Spectral and Thermal Imagery. Drones.

[10] Levin S., Yuan M., Adler M. Thermographic Quantification for Archaeological Prospection at Picuris Pueblo, New Mexico. Proceedings of the 3rd Digital Heritage International Congress.

[11] Moriarty C., Cowley D.C., Wade T., Nichol C.J. (2019). Deploying Multispectral Remote Sensing for Multi-temporal Analysis of Archaeological Crop Stress at Ravenshall, Fife, Scotland. Archaeol. Prospect..

[12] Brooke C., Clutterbuck B. (2019). Mapping Heterogeneous Buried Archaeological Features Using Multisensor Data from Unmanned Aerial Vehicles. Remote Sens..

[13] Uribe Agudo P., Angás J., Romeo F., Pérez-Cabello F., Santamaría D. (2021). Mapping Ancient Battlefields in a multi-scalar approach combining Drone Imagery and Geophysical Surveys: The Roman siege of the oppidum of Cabezo de Alcalá (Azaila, Spain). J. Cult. Herit..

[14] Abate N., Rubies D., Vitale V., Sileo M., Sogliani F., Masini N., Lasaponara R. (2022). Integrated use of multi-temporal multi-sensor and multiscale Remote Sensing data for the understanding of archaeological contexts: The case study of Metaponto, Basilicata. J. Phys. Conf. Ser..

[15] Masini N., Sogliani F., Sileo M., Abate N., Danese M., Vitale V., Lasaponara R., Piro S. (2022). Fusion and integration of heterogeneous close range remote sensing and geophysical data. The case of Grumentum. J. Phys. Conf. Ser..

[16] Blochin J.K., Pavlovskaia E.A., Sadykov T.R., Caspari G. (2023). Remotely Sensing the Invisible—Thermal and Magnetic Survey Data Integration for Landscape Archaeology. Remote Sens..

[17] Muscas L., Demontis R., Lorrai E.B., Heilmann Z., Satta G., Deidda G.P., Trogu A. (2024). Non-Invasive Survey Techniques to Study Nuragic Archaeological Sites: The Nanni Arrù Case Study (Sardinia, Italy). Geomatics.

[18] Verhoeven G.J. (2011). Near-Infrared Aerial Crop Mark Archaeology: From its Historical Use to Current Digital Implementations. J. Archaeol. Method Theory.

[19] Evans R., Jones R.J.A. (1977). Crop marks and soils at two archaeological sites in Britain. J. Archaeol. Sci..

[20] Materazzi F., Pacifici M. (2022). Archaeological Crop Marks Detection through Drone Multispectral Remote Sensing and Vegetation Indices: A New Approach Tested on the Italian Pre-Roman City of Veii. J. Archaeol. Sci. Rep..

[21] Materazzi F., Pacifici M., Santaga F.S. (2024). From Top to Bottom. Multispectral Remote Sensing and Data Integration to Rediscover Veii. FOLD&R FastiOnLine Doc. Res..

[22] Materazzi F. (2024). Per una Ricostruzione Dell’impianto Urbano di Veio. Dalla Ricerca Archivistica al Remote Sensing da Drone. Ph.D. Thesis.

[23] Riga F., Toso S., Genghini M., Carnevali L. (2009). Il problema dei danni da ungulati alle colture agroforestali. I Georg. Quaderni.

[24] Czajlik Z., Árvai M., Meészáros J., Nagy B., Rupnik L., Pásztor L. (2021). Cropmarks in Aerial Archaeology: New Lessons from an Old Story. Remote Sens..

[25] Biella M.C., Carlucci C., De Lucia Brolli M.A., Giuliani B., Lambiase L., Ligabue G., Materazzi F., Pacifici M. (2022). Falerii, Località Vignale. La Ripresa Delle Indagini in Un Settore Strategico Della Città Antica. Sci. Dell’antichità.

[26] Materazzi F. (2024). How Far Down? Interdisciplinary Discussions and Multimodal Investigations to Understand the Potential of Multispectral Remote Sensing. Proceedings.

[27] Biella M.C. (2024). Giving Voice to a Pre-Roman City: The Case of Falerii.

[28] Carlucci C., De Lucia Brolli M.A., Keay S., Millett M., Strutt K. (2007). An Archaeological Survey of the Faliscan Settlement at Vignale, Falerii Veteres (Province of Viterbo). Pap. Br. Sch. Rome.

[29] Hejcman M., Smrz Z. (2012). Cropmarks in stands of cereals, legumes and winter rape indicate sub-soil archaeological features in the agricultural landscape of Central Europe. Agric. Ecosyst. Environ..

[30] Agapiou A., Hadjimitsis D.G., Alexakis D.D. (2012). Evaluation of Broadband and Narrowband Vegetation Indices for the Identification of Archaeological Crop Marks. Remote Sens..

[31] Rouse J.W., Haas R.H., Schell J.A., Deering D.W. Monitoring Vegetation Systems in the Great Plains with ERTS. Proceedings of the 3rd ERTS-1 Symposium.

[32] Adamopoulos E., Rinaudo F. (2020). UAS-Based Archaeological Remote Sensing: Review. Drones.

[33] Geoportale Nazionale. http://www.pcn.minambiente.it/viewer/.

[34] Bell L.W. (2005). Relative growth rate, resource allocation and root morphology in the perennial legumes, Medicago sativa, Dorycnium rectum and D. hirsutum grown under controlled conditions. Plant Soil.

[35] Guaitoli M., Guaitoli M. (2003). Veio. Lo sguardo di Icaro, Le collezioni dell’Aerofototeca nazionale per la conoscenza del territorio.

[36] Casana J., Wiewel A., Cool A., Hill A.C., Fisher K.D., Laugier E.J. (2017). Archaeological Aerial Thermography in Theory and Practice. Adv. Archaeol. Pract..

